# Acceptance, Perceived Usefulness, and Data Sharing in Mobile Health Apps Among Patients With Breast Cancer: Cross-Sectional Survey Study

**DOI:** 10.2196/77750

**Published:** 2026-04-07

**Authors:** Madeleine Flaucher, Tabea Ott, Michael Nissen, Peter Dabrock, Hanna Huebner, Matthias W Beckmann, Peter A Fasching, Heike Leutheuser, Bjoern M Eskofier, Alexander Mocker

**Affiliations:** 1 Department Artificial Intelligence in Biomedical Engineering, Friedrich-Alexander-Universität Erlangen-Nürnberg, Nürnberger Straße 74, Erlangen, 91052, Germany; 2 Department of Ethics and Law in Medicine, University of Vienna and Medical University of Vienna, Austria; 3 Department of Systematic Theology and the Study of Religions, University of Vienna, Austria; 4 Department of Theology, Chair of Systematic Theology (Ethics), Friedrich-Alexander-Universität Erlangen-Nürnberg, Erlangen, Germany; 5 Department of Gynecology and Obstetrics, Universitätsklinikum Erlangen, Friedrich-Alexander-Universität Erlangen-Nürnberg, Erlangen, Germany; 6 Comprehensive Cancer Center Erlangen (CCC ER-EMN), Erlangen, Germany; 7 Comprehensive Cancer Center Alliance (CCC WERA), Erlangen, Germany; 8 Bavarian Cancer Research Center (BZKF), Erlangen, Germany; 9 Department of Computer Science, Chair of Machine Learning in Medicine, University of Bayreuth, Bayreuth, Germany; 10 Translational Digital Health Group, Institute of AI for Health, Helmholtz Zentrum München, Neuherberg, Germany

**Keywords:** mHealth applications, women's health, smartphone, digital health, data sharing

## Abstract

**Background:**

Mobile health (mHealth) apps promise to enhance patient empowerment, enable real-time health monitoring, and support self-management. Patients with breast cancer stand to benefit particularly from these capabilities, given the demanding pre- and posttreatment procedures they face. However, the effective adoption of these tools is challenged by issues including accessibility, usability limitations, and privacy concerns.

**Objective:**

This study aimed to investigate the current usage behavior and attitudes toward mHealth apps among patients with breast cancer in Germany, focusing on acceptance, usefulness, empowerment, and data-sharing. Specifically, we aimed to identify the incentives and barriers influencing use and the intention to use mHealth apps at the time of this writing to guide their further implementation and use.

**Methods:**

A cross-sectional online survey was conducted between November 2023 and June 2024 using a structured questionnaire developed in German. Participants with a self-reported breast cancer diagnosis were recruited via university hospital channels, patient associations, and social media. Topics included technology and health app usage, perceived usefulness, personal empowerment, and attitudes toward data sharing. The survey comprised both closed- and open-ended questions to capture quantitative patterns and qualitative insights into participants’ experiences and perspectives.

**Results:**

We collected responses from 90 participants with breast cancer. Participants demonstrated high engagement with digital technology; 97% (87/90) reported daily smartphone use, and 70% (63/90) owned a smartwatch or fitness tracker. Approximately 67% (60/90) of respondents had used health apps in the past year, with many relying on general fitness and lifestyle apps to support their health management. Most participants (62/90, 69%) indicated that health apps contribute to a better understanding of their personal health. Health apps were generally perceived as useful, with users highlighting their ability to provide continuous support and timely, reliable health information (n=31). This, in turn, was seen as instrumental in promoting self-management and empowerment. Respondents also showed a conditional willingness to share personal health data for research, particularly when clear benefits for treatment advancements were evident (n=82). Nonetheless, concerns were raised regarding data privacy, app usability, the need for multiple apps to cover all necessary features, and the potential increase in fear of illness (cyberchondria).

**Conclusions:**

The findings underscore the potential of mHealth apps to empower patients with breast cancer by improving health literacy and supporting self-management. To maximize their impact, future digital health tools must align closely with patient needs by incorporating adaptive, interactive features and integrating seamlessly with clinical care. Addressing key barriers—particularly data privacy and usability issues—is essential for broader adoption. Longitudinal studies are warranted to evaluate the long-term effects of health app usage on patient empowerment, quality of life, and clinical outcomes.

## Introduction

The intersection of technology and health care has introduced new pathways in mobile health (mHealth) apps. These digital tools promise enhanced patient autonomy, real-time health monitoring, and personalized care, capabilities particularly appealing for managing chronic diseases as well as long-lasting, complex, and potentially recurring conditions [1]. For patients with breast cancer, mHealth apps could represent a vital resource during their health treatment process [2,3].

Breast cancer is one of the most commonly diagnosed cancers among females worldwide, with approximately 2.3 million new cases annually [4-6]. The diverse nature of the disease requires targeted and individualized treatment [7]. Beyond the physical health implications, breast cancer impacts the emotional and psychological well-being of patients, often leading to anxiety, depression, and a reduced quality of life (QoL) [8]. These effects frequently extend to their families and support networks, who may experience emotional distress and caregiver burden [9]. Hence, effective management and care for breast cancer demand a holistic approach that incorporates medical, psychological, and social support to mitigate these effects [10,11].

By helping navigate the complexity of treatment, emotional distress, and long-term care, mHealth apps have great potential. The technology has the potential to help patients better understand their personal health status, contribute to self-management strategies, and thus improve health outcomes and QoL. Yet the promise of technological innovation often collides with realities of patient experience, where acceptance and adoption are shaped by more than functionality. On the one hand, existing solutions in women’s health available in app stores often lack trustworthy resources, a user-centered design with high usability, and research-supported effectiveness [12,13]. Some might even represent risks for patients through outdated health information. Apps developed within a thorough research process and evaluation, on the other hand, usually represent island solutions that were never made available to the general public [14].

Furthermore, existing literature primarily addresses technical and usability aspects, often overlooking the multifaceted needs of patients. While ensuring technical functionality and user-friendly design is critical, these aspects alone do not guarantee the effective integration of mHealth tools into the daily lives of patients. The development requires a comprehensive, patient-centered research approach that prioritizes emotional, cultural, and ethical considerations alongside technological development.

The primary aim of this survey study is to examine this usage behavior, acceptance, perceived usefulness, empowerment, and data-sharing attitudes related to mHealth apps among patients with breast cancer. Secondary objectives were to identify strategies to enhance adoption, engagement, and effective data sharing for future mHealth apps.

## Methods

### Survey Design

**Figure 1.**
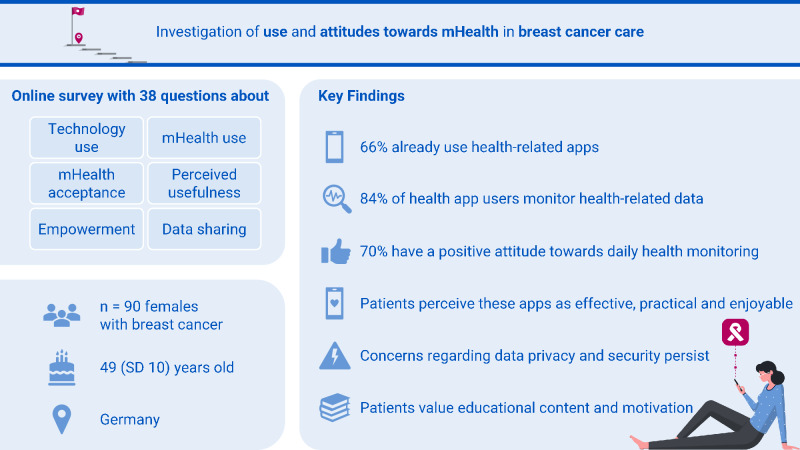
Visual abstract summarizing the objectives, methodology, and key findings of our survey study on health app usage among patients with breast cancer.

A questionnaire was designed to investigate several aspects related to the use of health apps among individuals with breast cancer. This study is embedded in the DigiOnKo project [15], an interdisciplinary initiative aimed at advancing digitally supported oncology care pathways by developing, evaluating, and implementing digital tools to support patients and clinical teams. An overview of the study is provided in Figure 1. Based on our research goal, we defined 2 exclusion criteria, individuals who had never been diagnosed with breast cancer in their lives or were under the age of 18 years. The questionnaire was designed in German. Besides basic demographic information, questions covered technology access, health app use, perceived usefulness of health apps, personal empowerment, health app acceptance, and data sharing. Further details about the survey instrument will be presented in the following chapters.

The survey was implemented in Unipark (Tivian XI GmbH). The questions included both open and closed questions; answer options were mainly on a 5-point Likert scale for closed questions. All items of the questionnaire were mandatory. All questions asking sensitive information provided a nonresponse option of “Prefer not to answer,” “not applicable,” or “neutral.” Participants were able to review all answers before finishing the survey. The question order was not randomized; 9 questions were conditional, depending on answers to previous questions.

The minimum number of items was 29 and the maximum number was 38. These questions were distributed over 21 survey pages in total. The full questionnaire can be found in Multimedia Appendix 1. Before submitting the questionnaire, an optional feedback field was provided for any further comments or remarks. The survey included 6 open questions, where participants were instructed to provide short written answers in bullet points. Additionally, a text field for further comments and feedback was provided.

#### Technology and Health App Use

To gain an overview of the usage of technology in general and related to their personal health, all participants were asked about the use and usage frequency of smartphones, tablets, and wearables. The aim was to gain insights into the engagement and adoption of technology in general and mHealth in particular. This was complemented by questions regarding internet usage frequency and the confidence in using smartphones and mobile apps.

Related to personal health, participants were asked about recently used resources to improve their personal health (podcasts and audiobooks, social media, video calls, smartphone apps, printed materials, and internet). Specifically, we asked which health apps they have installed on their devices, how often they use them, what they use them for, and whether they felt informed about the risks and benefits associated with these apps at the time of this writing. The question was targeted specifically at all health-related apps and was not limited to specific breast cancer apps, as patients might use a variety of different apps to manage their condition (eg, general fitness apps or meditation apps).

#### Perceived Usefulness

According to the Technology Acceptance Model (TAM), the perceived usefulness of a technology is closely linked to their attitude toward it as well as their intention to use it [16]. If patients perceive mHealth apps as useful, they are more likely to engage with them regularly. As shown in a meta-analysis by Gan et al [17], greater engagement in mHealth apps was significantly associated with mental health improvements. Perceived usefulness was measured through a questionnaire developed by Voss et al [18] that has been used in previous work and captures the hedonic and utilitarian dimensions of attitudes toward a product category. With 10 different adjective pairs, it captures the perceived usefulness on a 5-point Likert scale, for example, effective-not effective and boring-exciting. Additionally, participants were asked about the perceived benefits and risks in 2 open questions.

#### Personal Empowerment

Patient empowerment is fundamental for effective chronic disease management, as it is closely linked to improved self-management, better adherence to treatment, and enhanced overall well-being. Empowered patients are more likely to engage in informed decision-making, feel confident in managing their condition, and experience greater autonomy in their care journey [19,20]. In this study, all measurement items were adapted from Lemire et al [21] and included items related to the ability to apply gained knowledge to effectively make decisions related to personal health [22]. To further explore this dimension, participants were also asked whether they experience discrimination in their daily lives and whether they fear additional disadvantages or discrimination related to using mHealth apps.

#### Acceptance of Health Apps

Daily interaction with an mHealth app can induce psychological harm from health anxiety or cyberchondria, the excessive or compulsive seeking of online health-related information that exacerbates anxiety [23,24]. For patients with breast cancer, this phenomenon can be particularly pronounced, as apps often encourage users to continuously monitor their health, presenting daily reminders of their condition.

To investigate the acceptance toward health apps in breast cancer care, we used questions from Potdar et al [25] that have been used to investigate acceptability toward mHealth among patients with cancer in general. The questions targeted features for monitoring the health status, tracking information on the smartphone, and using a health app to improve their health-related knowledge, as these represent features often used in mHealth apps for breast cancer [26]. With an open question, we then asked participants about specific features they would expect or want in an mHealth app designed to support patients with breast cancer.

#### Data Sharing

According to the Privacy Calculus Theory, individuals weigh perceived benefits, such as improved therapies or valuable research contributions, against potential risks, like loss of privacy or misuse of data [27]. By targeting data-sharing scenarios under specific conditions—such as offering incentives, ensuring transparency in data use, or highlighting therapeutic advancements—we aimed to identify the factors that encourage or discourage data sharing. For this purpose, we used an adapted version of the questionnaire developed by Pletscher et al [28]. Questions targeted specifically at sharing data with research institutions under certain conditions, for example, if incentives are offered, therapies can be improved, or the purpose for the data use is known. Additionally, reasons why participants would want to share or not share their data were asked in an open question.

### Survey Pretesting

The final questionnaire went through a test run with 4 independent assessors to identify any usability issues, verify that questions are clear and understandable, and reduce any possible biases in questions.

### Recruitment Process

The survey was designed as an open, online-only survey; everyone with access to the link was able to participate on a voluntary basis. On the landing page of the survey, website visitors were informed about the study, the objectives, the estimated duration, the types of data collected within the survey, storage of the data, and the contact information of the investigators in charge. In order to start the survey, active consent for participation was required. After consent, 2 eligibility questions were asked; all participants had to be at least 18 years old and had to have a breast cancer diagnosis at some point in their lives, as the target population was patients with breast cancer.

A convenience sampling strategy was chosen. The survey was distributed through a combination of clinic-based recruitment and online channels. The survey was promoted by posters and flyers in the university hospitals in Erlangen and Würzburg (Bavaria, Germany); via personal contacts; as well as various mailing lists and patient associations. Online recruitment included the university website and social networks such as Instagram (Meta Platforms), Facebook (Meta Platforms), LinkedIn (Microsoft Corp), and nebenan.de (Good Hood GmbH). On Facebook, the survey was posted in patient groups related to breast cancer. On Instagram, various influencers were asked to distribute the flyer to their followers. Among them, PINK gegen Brustkrebs GmbH (Germany), the company behind the breast cancer app Pink! Coach promoted the survey through an advertisement. Social media recruitment was included to increase reach and accessibility, allowing participation from patients who might not regularly attend the recruiting clinics or who live outside the immediate hospital regions. The data collection was performed between November 2023 and June 2024.

### Data Analysis

Quantitative data were analyzed using descriptive statistics (frequencies, percentages, means, and SDs). All analyses were conducted using Python (Python Software Foundation). All answers to open questions were analyzed with an inductive category formation of a qualitative content analysis according to Mayring and Fenzl [29]. The analysis was conducted following Mayring’s systematic procedure of iterative category development; initial codes were developed and refined through repeated review. Representative quotes were selected to illustrate key themes. Survey results are reported in accordance with the CHERRIES (Checklist for Reporting Results of Internet E-Surveys) [30]; the full checklist is provided in Checklist 1.

### Ethical Considerations

All data were collected anonymously. The dataset did not contain any personal data in accordance with the GDPR. Thus, this study was exempt from institutional review. The exemption was confirmed by the Institutional Review Board of FAU Erlangen-Nürnberg (23‐333-ANF). Informed consent was obtained from all participants prior to participation in the survey; participants did not receive any compensation.

## Results

### Survey Statistics

In total, the landing page of the survey was visited 404 times, and 154 individuals agreed to participate in the survey and filled out the eligibility screening. 145 reportedly met the defined criteria and started the questionnaire, and 90 completed it. Therefore, the completion rate was 62%. Only completed surveys are included in the subsequent analysis. On average, participants spent 15.9 (SD 7.8) minutes completing the survey. No atypical timestamps were detected; therefore, no participant was excluded due to the duration of the participation.

### Participant Demographics

The mean age of participants is 49 (SD 10) years, with 88 out of 90 (98%) indicating female sex; 2 (2%) participants out of 90 preferred not to specify. Detailed sociodemographic information about the participants is shown in Table 1.

**Table 1.** Demographic information of all survey participants.

Demographics	Participants (N=90)
Age (years)	
Range	26-71
Mean (SD)	49 (10)
Age at breast cancer diagnosis (years)	
Range	25-69
Mean (SD)	49 (9)
Gender, n (%)	
Woman	88 (98)
Unspecified	2 (2)
Education, n (%)	
High school diploma	33 (37)
Vocational education	26 (29)
University degree	27 (30)
Doctoral degree	1 (1)
Unspecified, n (%)	3 (3)
Employment, n (%)	
Full-time	30 (33)
Part-time	33 (37)
Self-employed	1 (1)
Retired	13 (14)
Nonworking	7 (8)
Unspecified	6 (7)
Health insurance, n (%)	
Private	14 (16)
Statutory	75 (83)
Unspecified	1 (1)
Cancer therapy status, n (%)	
In therapy	48 (53)
Aftercare	40 (44)
Unspecified	2 (2)

### Technology Usage

All participants possess a smartphone, and most use it daily (87/90, 97%). A majority (63/90, 70%) have a smartwatch or fitness tracker and use it daily or almost daily (52/90, 83%). Additionally, participants rated how confident they feel using smartphones and apps. A majority feels very confident (46/90, 51%) or rather confident (26/90, 29%); a few participants feel very insecure (4/90, 4%) or rather insecure (2/90, 2%) in smartphone and app use. The majority of participants (86/90, 86%) reported a daily use of the internet; all participants accessed the internet at least once a week.

### mHealth Usage

Among all participants, 59/90 (66%) stated that they had used mobile apps to improve their personal health status within the last 12 months. The internet in general was used by 82 (91%), social media by 72 (80%), and audiobooks or podcasts by 51 (57%).

When asked about installed apps on their devices, 70 (78%) participants listed the apps. On average, participants had 2 apps related to their health installed; one participant listed 15 health-related apps.

The most frequently mentioned app was “Pink! Coach” (25/90, 28%), a medically certified app in Germany specifically designed to improve QoL and health literacy in patients with breast cancer [31]. Besides that, general fitness apps such as Fitbit (Fitbit Inc), Garmin Connect (Garmin Ltd), or Apple Health (Apple Inc) are frequently installed.

Regarding the frequency of using these apps, 59 out of 90 (66%) stated at least once a week, and 21 out of 90 (23%) mentioned daily use. Among all participants (n=90), 14 (16%) reported that they are not using health apps. A filter was applied so that only those who indicated app usage (n=76) received the subsequent questions regarding app usage purpose.

**Figure 2.**
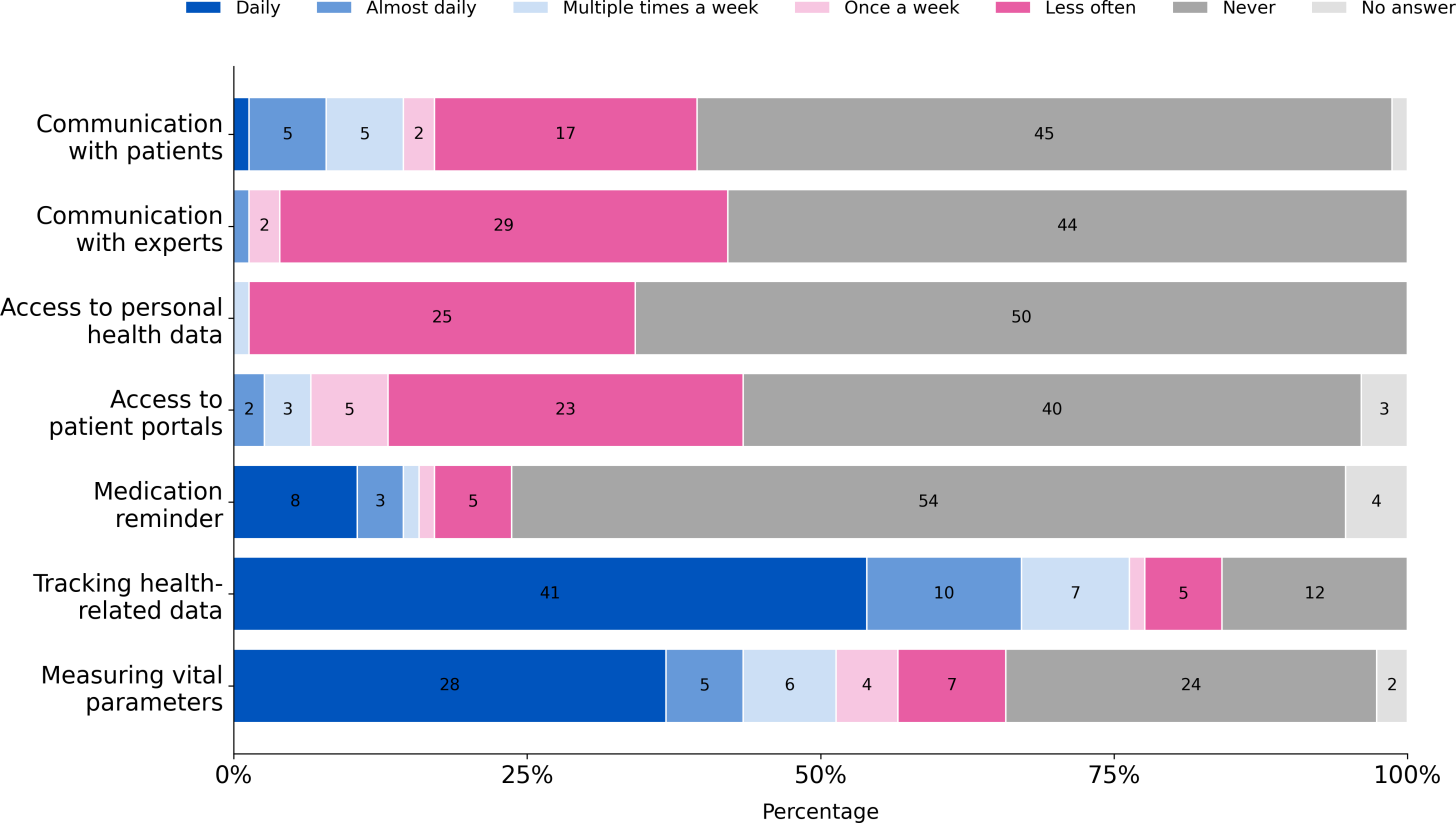
Answer distributions of the usage frequency to different features commonly included in mHealth apps by participants who use health apps (n=76).

The purpose and frequency of using these apps for specific purposes are represented in Figure 2. Most participants use their apps to measure physiological parameters such as heart rate and to monitor other health-related data such as physical activity, steps, or diet. As shown in Figure 2, 67% (n=51) of app users are already monitoring health-related parameters daily or almost daily. In contrast, the use of an electronic health record, contact with health professionals or other patients, and access to patient portals were not common among the participants.

Among the app users, participants were asked whether they had been informed about the benefits and risks of using the health app before starting. A total of 30 (40%) out of 76 app users reported feeling uninformed about the risks associated with the app. In contrast, 36 out of 76 (47%) stated they had been adequately informed about their benefits.

### Acceptance

**Figure 3.**
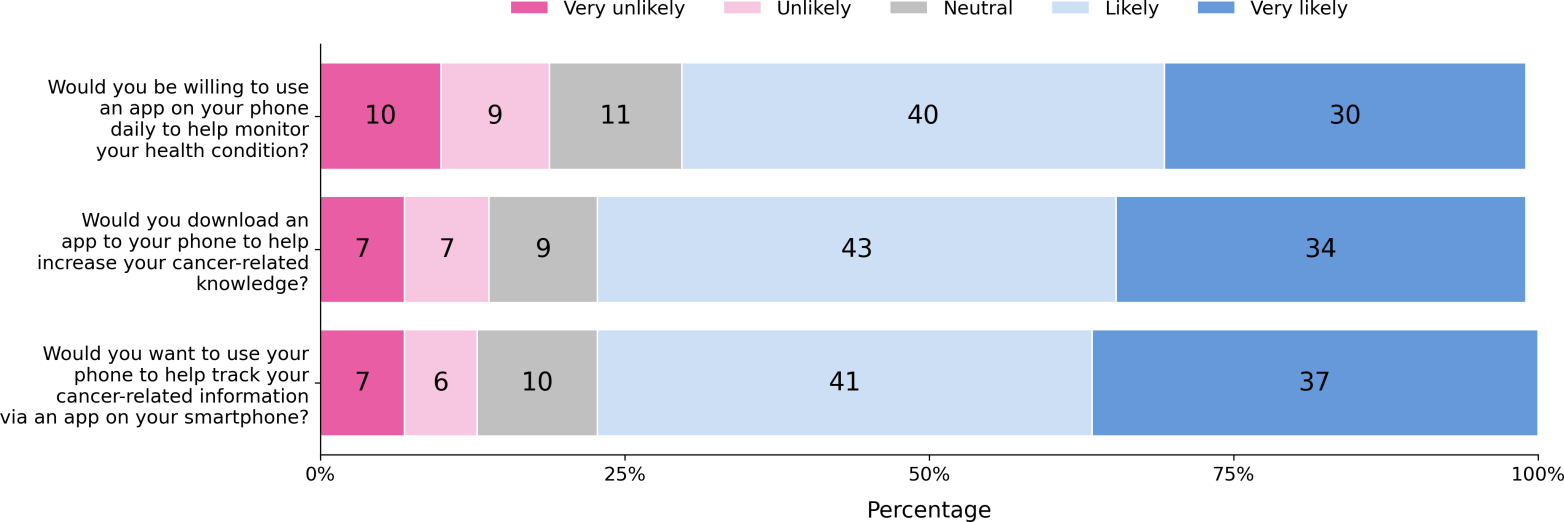
Answers given to the acceptance-related survey items in percentages.

Figure 3 shows the answers of all participants to the 3 questions related to the general acceptance of cancer-related mobile apps. Most participants are willing to use their smartphone for tracking cancer-related information through a mobile app and to use it as a health monitor on a daily basis. Furthermore, a majority of participants would be willing to download a mobile app to increase their knowledge about cancer.

### Perceived Usefulness

**Figure 4.**
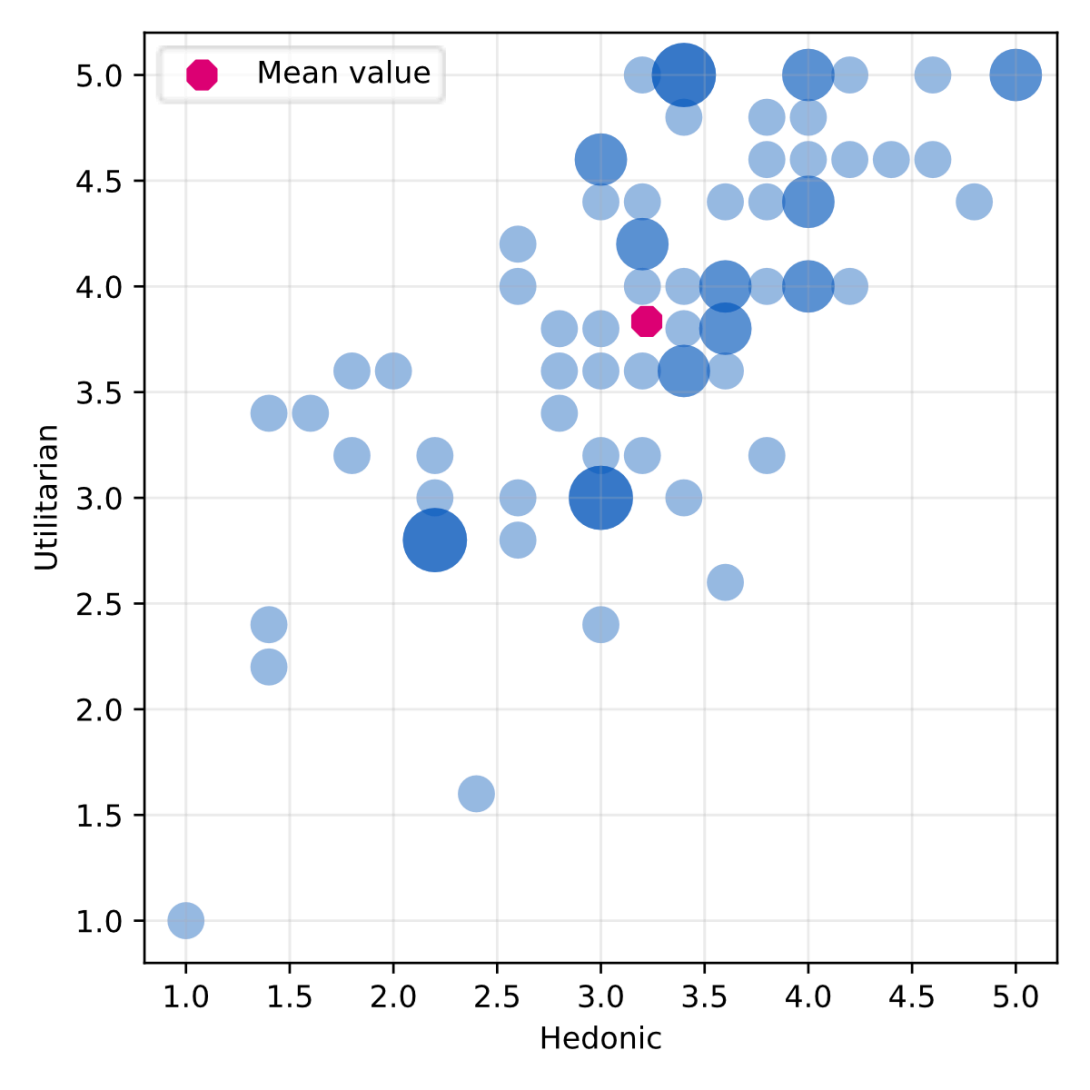
Scatterplot illustrating perceived usefulness scores for all participants (blue), where larger circles indicate multiple participants with the same response. The overall average is highlighted in pink.

The utilitarian and hedonic dimensions of the attitude toward health apps of users are shown in Figure 4. Overall, the average on the hedonic dimension was 3.22 (SD 0.87) and on the utilitarian dimension 3.83 (SD 0.87). Thus, the used mHealth apps are perceived as both pleasurable and useful.

**Figure 5.**
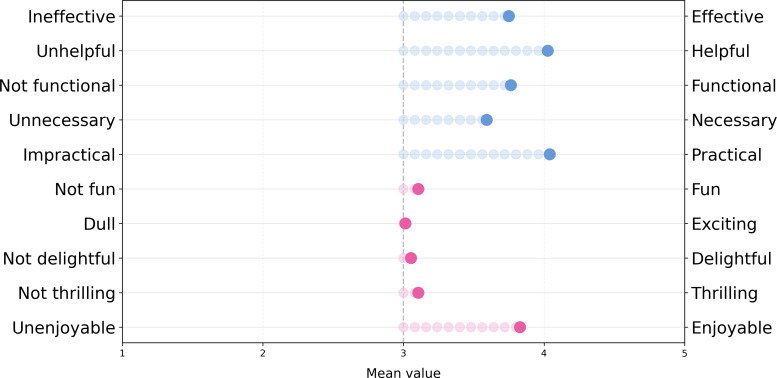
Mean values for all categories of the utilitarian and hedonic dimensions of the questionnaire for perceived usefulness.

In more detail, as shown in Figure 5, participants perceive their apps as effective, practical, and enjoyable.

### Personal Empowerment

**Figure 6.**
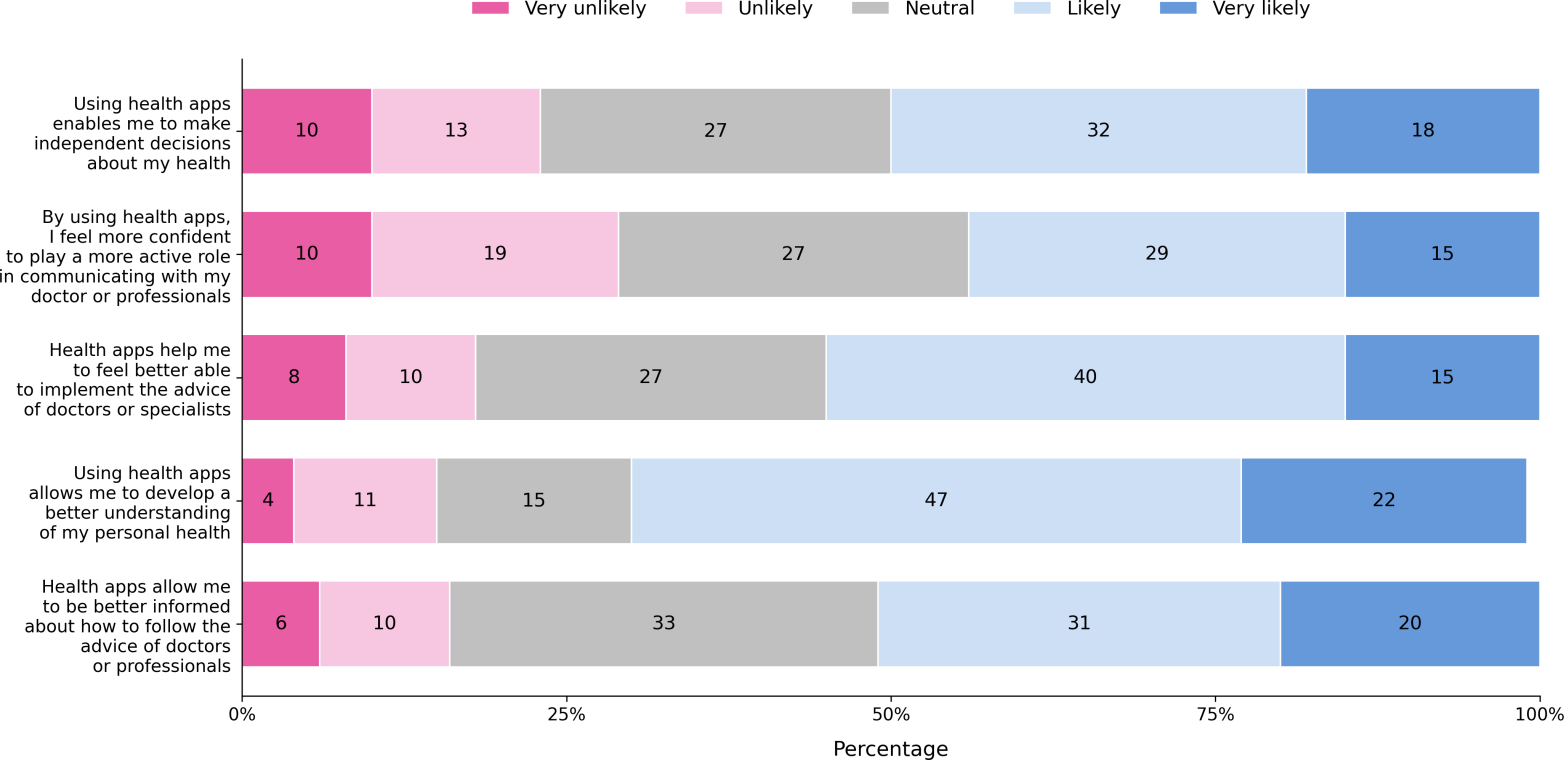
Participants’ responses to 5 different aspects of personal empowerment: (1) making independent health decisions, (2) engaging more actively with health care professionals, (3) feeling more capable of following medical advice, (4) gaining a better understanding of personal health, and (5) staying informed on how to follow professional recommendations.

The findings, as illustrated in Figure 6, reveal varying levels of agreement among participants regarding the empowering potential of health apps. A majority of respondents indicated that health apps likely or very likely enable them to develop a better understanding of their personal health (62/90, 69%) and implement advice from doctors or specialists (49/90, 54%). Similarly, 39 out of 90 (43%) of participants felt that using health apps enhances their confidence in communicating with health care professionals, while 45 out of 90 (50%) agreed that these tools help them make independent decisions about their health. Overall, a minority of participants perceived health apps as very unlikely or unlikely to contribute to these empowerment domains. A total of 29% (26/90) think it is unlikely that using health apps could give them a more active role when talking with their doctors. With an overall mean for all items of 3.4 out of 5.0, the participants report a positive contribution of health apps to personal empowerment.

### Data Sharing

**Figure 7.**
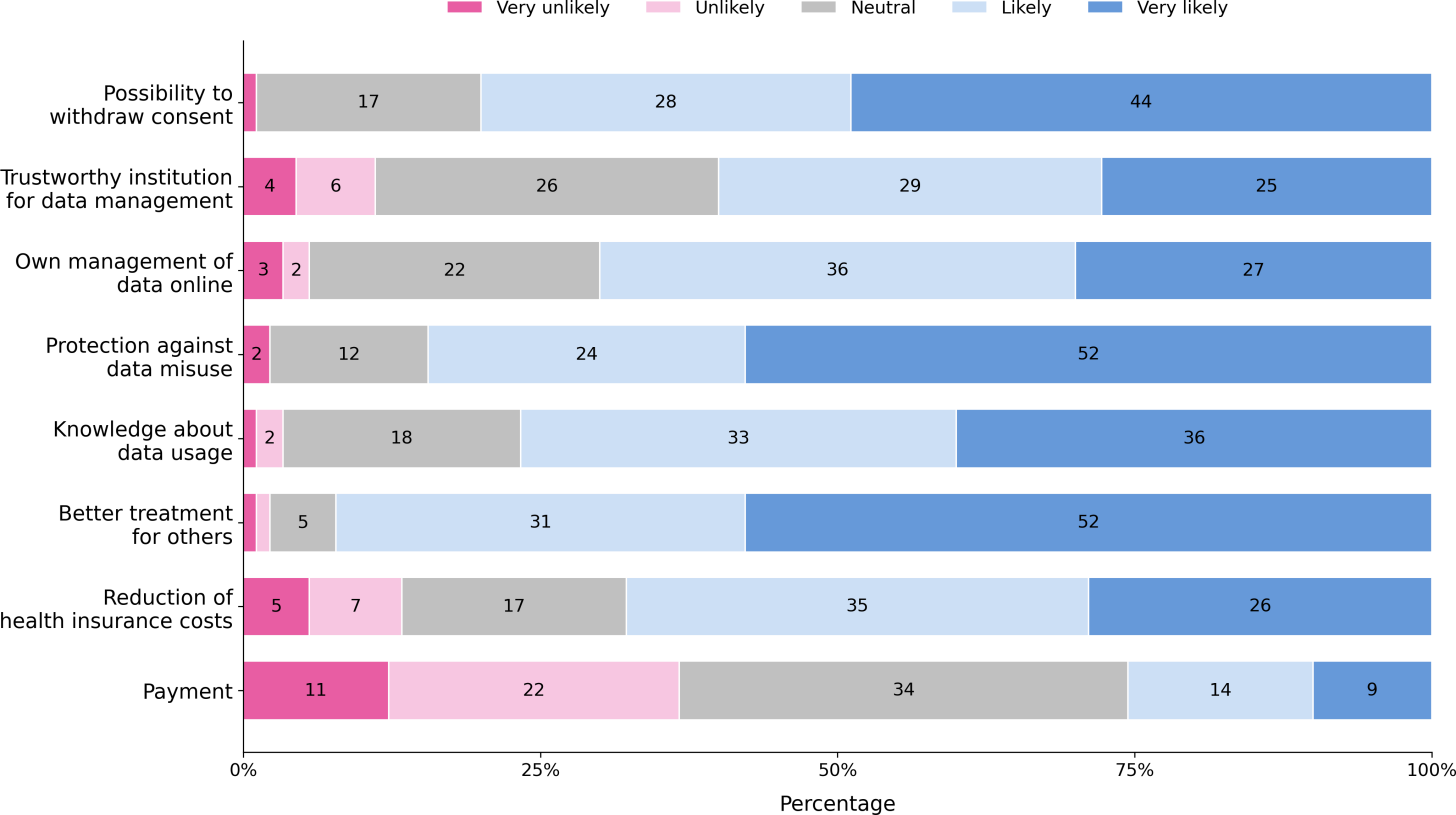
Suggested incentives and the participants’ attitudes toward sharing personal health data for research purposes.

The most relevant drivers of sharing personal health data for research purposes from the list provided to the participants, as shown in Figure 7, are (1) if people knew that by doing so, others would receive a better treatment (n=82 likely, or very likely); (2) if people were sure that data were protected from misuse (n=75 likely, or very likely); (3) if people could withdraw from the consent of data usage anytime (n=72, 80%); and (4) if it were known what research is done with the data (68/90, 76%). On the other hand, money was perceived as less of an incentive (22/90, 24% likely or very likely).

**Figure 8.**
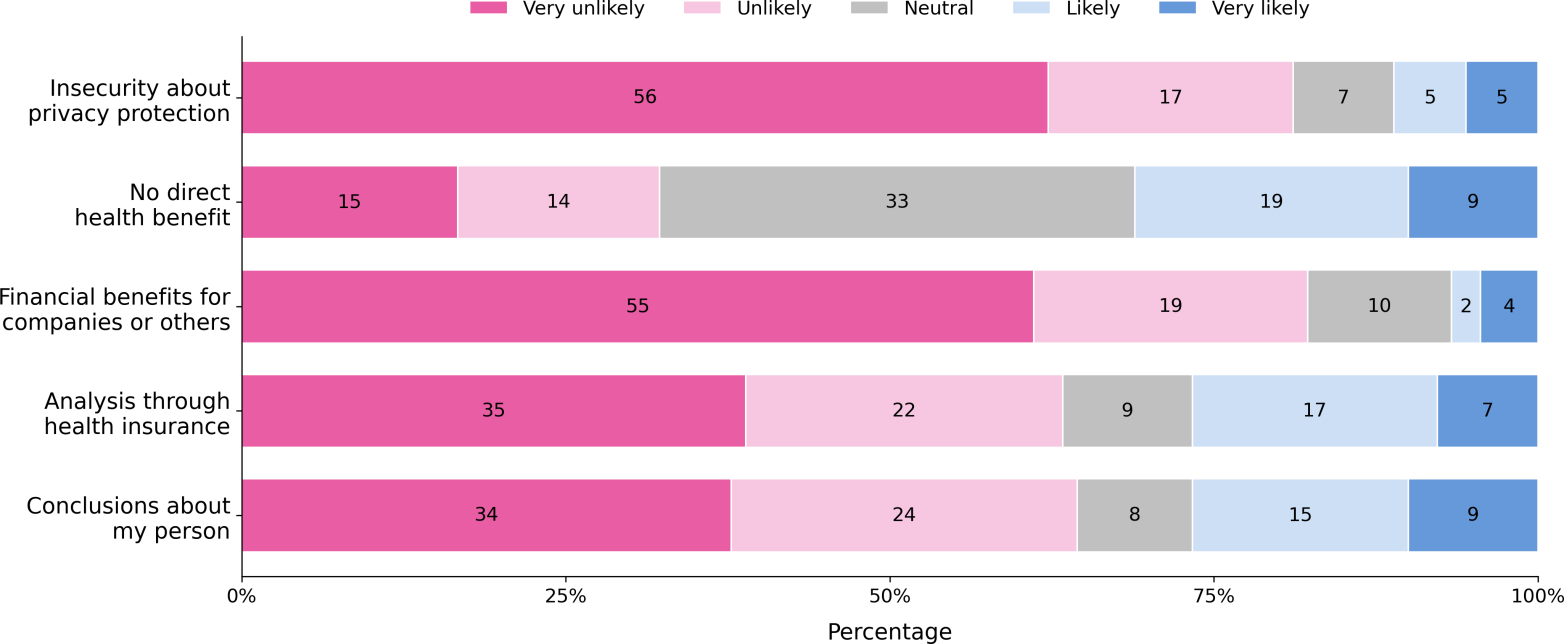
Attitudes of participants toward 5 barriers to sharing personal health data.

Concerns about privacy protection and financial benefits for companies were the most significant barriers, with the majority of respondents indicating these were “Very likely” to hinder their data sharing. A lack of direct health benefit is perceived as less of a barrier. A detailed overview of the responses to potential barriers is shown in Figure 8.

### Qualitative Data Analysis

#### Required App Features and Content

Participants were asked to list specific features they expect in an mHealth app supporting patients with breast cancer. Related to general usability and design features, 6 main categories were identified. Mostly mentioned were notifications and reminders (n=17), followed by clearness and simplicity (n=9), motivational features (n=7), adaptation to individual needs (n=6), and trustworthiness (n=4).

In terms of educational content, in addition to general cancer-related information (n=58), detailed advice and recommendations on diet (n=20), physical activity (n=19), and topics related to mental health and general well-being (n=17) were of particular interest to participants. In addition, more updates on recent research results and ongoing studies were frequently requested (n=16). Participants emphasized the importance of up-to-date information that evolves over time (“Up-to-dateness, continuous expansion of information” P79, translated), as well as concise explanations of medical terms (“Database [Explanation of terminology]” P69, translated).

#### Benefits and Risks of mHealth Apps

When asked about the benefits mHealth apps offer in the context of breast cancer, 31 specifically mentioned the provision of information, especially up-to-date information (n=2) and trustworthy information (n=2). Furthermore, participants see benefits in motivating users (n=20) to eat healthy or exercise. A large group of participants (n=22) highlighted the personal and mental support of these apps in dealing with disease, therapy, and side effects. Especially since they are available 24 hours a day (n=5). For example, one participant mentioned that these apps offer

*personal support and helpful input, that can be selected individually, in addition to the often limited time available during consultation hours*

(P33, translated)

Participants also mentioned that these apps can provide a sense of control and security (n=7). For example, they could help to

*assess your personal lifestyle and identify opportunities for improvement (gives you the confidence to “do something” yourself)*

(P33, translated)

In contrast, 23 participants did not see any benefits in health apps. As reasons, participants mentioned, for example, a lack of knowledge about available apps (“I would like more information about the available options,” P36, translated) or that information is already available on the internet (“This knowledge can be obtained on the Internet on your own,” P23). One participant stated that, in their experience, mHealth apps usually cover only a small aspect, lack usability, and are difficult to activate due to the need for a doctor’s prescription (P90).

When asked about the risks of mHealth apps, the majority of participants (n=42) stated that they did not see any risks. Only 10 participants expressed concerns regarding data privacy and security. Furthermore, a few participants were concerned about potential misinformation (n=8) or negative mental health effects (n=6), for example, by providing wrong diagnoses or untrustworthy sources. Additionally, 7 participants mentioned a potential overload, for example, through providing too much information. As obstacles hindering participants in using available health apps, participants mentioned reasons such as insufficient quality, lack of usability, lack of time, or lack of cost coverage by health insurance.

## Discussion

### Overview

With this study, we investigated self-reported usage patterns, health app acceptance, and views on data sharing among patients with breast cancer in Germany. Our results provide insights into patients’ perspectives on mHealth technology, including aspects they perceive as beneficial as well as challenges and barriers that still need to be overcome. Especially, aspects related to usability, effectiveness, and privacy concerns need further attention from the patient’s viewpoint.

Participants reported a high prevalence of fitness tracker and smartwatch usage. This could have several reasons; on the one hand, this survey might have been especially interesting to technology-affine people. On the other hand, the popularity of mobile and wearable technology, particularly among older adults, is increasing [32]. Additionally, one of 6 patients with breast cancer in Germany is younger than 50 years old, which could also contribute to higher adoption of digital health tools in our sample [33,34].

In general, the majority of participants expressed openness to using mHealth apps for monitoring their personal health status and enhancing their health knowledge. Also, daily interaction with an app appears to be feasible and not a deterrent for most participants. This positive attitude aligns with the growing trend of integrating technology into health care practices, particularly for older adults [32].

### Health Apps as a Tool for Patient Empowerment

The findings suggest that health apps are generally perceived as useful and have the potential to empower patients. Participants specifically highlighted the availability of these apps, offering support around the clock in addition to limited interaction time with health care providers. This availability was mentioned as a key factor in fostering a sense of control and confidence, both of which are central to patient empowerment [35]. Participants valued apps that could deliver timely and reliable health-related guidance, highlighting a broader shift toward digital health literacy as a central component of modern patient care.

When designed effectively, such apps can facilitate quick and reliable answers to users’ questions, thereby fostering health literacy and eHealth literacy. This, in turn, has the potential to enhance self-management and improve QoL by supporting informed decision-making, healthy behaviors, and adherence to therapy. Notably, the extent to which these apps actually enhance self-management likely depends on factors such as usability, personalization, and integration with clinical care issues that remain underexplored in the digital health landscape at the time of this writing [36].

Effective empowerment requires not just access to information but also actionable, tailored guidance that aligns with individual health needs. To maximize their impact, future health apps should go beyond providing generic health content and instead offer dynamic, interactive features that support patient autonomy. This includes adaptive recommendations, such as tailored exercise or lifestyle guidance and nudges based on app usage patterns, but also the seamless integration with clinical care, for example, through secure messaging, automated report sharing, or appointment planning. Beyond these, user-driven customization options should be provided, such as adjustable dashboards, modular feature selection, and flexible notification settings. These features can help patients tailor the app experience to their individual needs, literacy levels, and desired depth of information and engagement. Further research is needed to identify which app functionalities most effectively contribute to different dimensions of empowerment and how these functionalities are perceived with different patient characteristics and care contexts.

### Widespread Use of Health Apps and Their Limitations

Interest in health apps is high, with many participants using apps not specifically designed for breast cancer management. Fitness apps, in particular, were frequently mentioned, as they support a healthy lifestyle, a key factor for many participants in improving QoL. This suggests that patients are already adopting existing digital tools to support their health, even in the absence of condition-specific alternatives. Furthermore, these findings suggest that patients exhibit intrinsic motivation to enhance their health and actively contribute to their therapy. It highlights their desire to play an active role in their health care journey.

However, while general health apps offer broad functionality, they may not address the unique concerns of patients with breast cancer, such as symptom tracking, treatment side effects, or emotional well-being. This raises an important question. Do patients actually need breast cancer-specific apps, or are they sufficiently served by more general health applications? The widespread use of existing tools suggests that some needs may already be met; however, it remains unclear whether these apps are fully adequate for the challenges of managing a chronic illness. Future research should investigate whether condition-specific features—such as integration with oncology care, personalized symptom management, or peer support—would enhance engagement and health outcomes. Understanding these preferences could inform the development of more targeted digital health solutions that complement, rather than duplicate, existing app functionalities.

### Balancing Data Privacy Concerns and Willingness to Share

Data privacy remains a central concern for many participants, with uncertainty surrounding how health apps handle sensitive personal information. This skepticism reflects broader public debates on digital health security and trust in technology providers [37]. However, our findings reveal that privacy concerns do not translate into outright rejection of data sharing. Instead, participants demonstrated a conditional willingness to share their health data, particularly when contributing to research with tangible benefits for future treatment advancements. This highlights a critical balance between risk perception and perceived utility, suggesting that concerns about data security are not absolute barriers but negotiable factors. These findings align with previous research indicating that patients with cancer are particularly open to data sharing when they perceive benefits for themselves or others from the research conducted [38].

Transparent communication regarding data usage, robust security measures, and opportunities for users to maintain control over their data are essential for improving trust. Beyond technical safeguards, actively involving patients in research processes—through co-design initiatives, participatory data governance models, or better communication of research findings—could further shift perceptions, positioning data sharing as an informed and mutually beneficial decision rather than a passive risk.

### Structural Barriers

Despite their potential, these health apps often fail to fully meet users’ needs. A key challenge was the fragmentation of app functionalities, with users often having to rely on multiple apps to cover different aspects of their health management. This lack of integration creates inefficiencies and may discourage long-term use. Additionally, structural issues—such as unclear reimbursement policies and prescription processes—further complicate the adoption of digital health solutions within formal health care systems. Additionally, our study did not account for individuals who have no structural or material access to mHealth at all, whether due to a lack of technical infrastructure (ie, no smartphone) or knowledge barriers (eg, illiteracy, lack of digital literacy).

### Psychological Impact of Health Tracking

Another notable concern was the psychological impact of continuous health tracking. Some participants questioned whether constant app engagement might lead to an excessive preoccupation with their illness rather than providing reassurance. This highlights the need for a balanced approach in app design. Digital tools should support patient autonomy without reinforcing anxiety or over-monitoring. While tracking tools can facilitate behavior change, their effectiveness depends on sustained engagement [39,40]. If apps demand too much attention or become emotionally taxing, users may abandon them over time. Striking the right balance between engagement and cognitive load is crucial for long-term use. Practical design strategies could include adjustable frequencies of self-monitoring, optional data summaries, or modular features that can be enabled or disabled depending on individual needs and preferences.

### Toward Integrated and Patient-Centered Solutions

Addressing these challenges requires a more holistic approach to app development. This approach to app development, characterized by fragmented and often redundant solutions, may not be sustainable [41]. Developers should shift their focus toward creating integrated, multifunctional platforms that address a broad spectrum of patient needs, as also identified in other related works [42,43]. Additionally, engaging stakeholders—including health care providers and patients—in the design process can help ensure that apps are practical, trustworthy, and aligned with real-world usage contexts [44]. Beyond design considerations, clear regulatory frameworks, standardized evaluation criteria, and improved access to reimbursement will be crucial in embedding digital health tools into routine care.

### Limitations

Overall, this work provides insights into the actual usage and perspectives toward mHealth apps among patients with breast cancer in Germany. Nevertheless, this study is subject to several limitations. The sample size was relatively small, which may limit the generalizability of the findings. The study was conducted as an online survey and was mainly distributed online. Thus, only participants with internet access and at least a computer or smartphone were able to participate. In combination with the general topic of the survey, it is likely that people with an affinity for technology in particular were addressed and that the group of patients without internet access is not represented in the collective. Due to the survey tool, which did not include cookies, internet protocol address checks, or other measures, repeated participation cannot be ruled out completely.

Since this study investigated self-reported data, actual usage patterns may differ from what participants stated in this questionnaire.

Furthermore, the survey was available only in German. As other countries have different possibilities of mHealth in breast cancer and are at different levels of digitizing health care, acceptance and usage might differ largely.

Furthermore, the participants were, on average, younger than the typical age at breast cancer diagnosis and had a higher level of education, which may limit the generalizability of the findings to the broader patient population. Finally, the small sample size and the fact that we were not able to recruit any non-female participants, even though specific organizations were contacted for that, highlight the need for interpreting the results carefully.

### Conclusions

This study provides valuable insights into current usage behaviors and attitudes toward health apps from the perspective of patients with breast cancer. We highlight key aspects that could enhance adoption, engagement, empowerment, and effective data sharing for future mHealth applications and research. Despite the lack of condition-specific apps, patients are already using general health and fitness apps to improve their QoL, indicating a strong intrinsic motivation for self-management in our study population. There is an opportunity to build on existing app usage behaviors by tailoring future digital health tools to meet the unique needs of patients with cancer. However, significant barriers, particularly privacy concerns, must be addressed to enhance trust and encourage broader adoption.

The findings emphasize the importance of aligning health apps with patient needs and preferences to maximize their utility and adoption. For health care, integrating apps into routine care pathways can provide patients with supplementary tools for self-management, potentially reducing the burden on health care providers. Collaboration among developers, health care professionals, and patients will be critical to ensure that future apps effectively address real-world challenges.

Further research is needed to assess the long-term impact of health apps on patient empowerment, including sustained engagement, decision-making autonomy, and self-efficacy. Additionally, studies should examine their effects on QoL, including mental well-being, treatment-related distress, and daily functioning, as well as clinical outcomes such as symptom management, adherence to therapy, and disease progression. Finally, the potential of artificial intelligence, machine learning, and wearable sensors should be explored to address the requirements of patients, such as personalization or real-time health insights.

## Supplementary material

10.2196/77750 Multimedia Appendix 1 Full version of the questionnaire in German.

10.2196/77750 Multimedia Appendix 2 English translation of the questionnaire.

10.2196/77750 Checklist 1 Full Checklist for Reporting Results of Internet E-Surveys (CHERRIES).

## Abbreviations

CHERRIES: Checklist for Reporting Results of Internet E-Surveys
mHealth: mobile health
QoL: quality of life
