# Supplementary material for: Acceptance, Perceived Usefulness, and Data Sharing in Mobile Health Apps Among Patients With Breast Cancer: Cross-Sectional Survey Study
Source: JMIR Cancer. 2026 Apr 7;12:e77750. doi: 10.2196/77750 (PMC13056039; doi:10.2196/77750)
Supplement: Multimedia Appendix 1 [file cancer-v12-e77750-s001.pdf]

digiOnko

In welchem Jahr wurden Sie geboren?

___

Wurde bei Ihnen jemals eine Brustkrebserkrankung festgestellt?

- ◯ Nein
- ◯ Ja

Besitzen Sie ein Smartphone?

- ◯ Nein
- ◯ Ja
- ◯ Keine Angabe

Besitzen Sie ein Tablet?

- ◯ Nein
- ◯ Ja
- ◯ Keine Angabe

Besitzen Sie eine Smartwatch oder einen Fitnesstracker?

- ◯ Nein
- ◯ Ja
- ◯ Keine Angabe
- ◯ Ich weiß nicht, was das ist

digiOnko

Wie häufig gehen Sie ins Internet?

- ◯ Täglich
- ◯ Fast täglich
- ◯ Mehrmals pro Woche
- ◯ Einmal pro Woche
- ◯ Seltener
- ◯ Keine Angabe

Wie sicher fühlen Sie sich im Umgang mit dem Smartphone und der Nutzung von Apps?

- ◯ Sehr unsicher
- ◯ Eher unsicher
- ◯ Neutral
- ◯ Eher sicher
- ◯ Sehr sicher
- ◯ Ich nutze kein Smartphone

Wie häufig verwenden Sie ihr Smartphone?

- ◯ Täglich
- ◯ Fast täglich
- ◯ Mehrmals pro Woche
- ◯ Einmal pro Woche
- ◯ Seltener
- ◯ Keine Angabe

Wie häufig verwenden Sie ihr Tablet?

- ◯ Täglich
- ◯ Fast täglich
- ◯ Mehrmals pro Woche
- ◯ Einmal pro Woche
- ◯ Seltener
- ◯ Keine Angabe

Wie häufig tragen Sie ihre Smartwatch oder Ihren Fitnesstracker?

- ◯ Täglich
- ◯ Fast täglich
- ◯ Mehrmals pro Woche
- ◯ Einmal pro Woche
- ◯ Seltener
- ◯ Keine Angabe

Haben Sie in den vergangenen 12 Monaten eine der folgenden Ressourcen genutzt, um Ihren Gesundheitszustand zu verbessern?

	Nein	Ja	Keine Angabe
Hörbücher und Podcasts	◯	◯	◯
Social Media (z.B. Facebook, Instagram, etc.)	◯	◯	◯
Video-Telefonate	◯	◯	◯
Apps für Smartphone und/oder Tablets	◯	◯	◯
Gedruckte Materialien (z.B. Flyer, Bücher, etc.)	◯	◯	◯
Internet (z.B. Google)	◯	◯	◯

digiOnko

Welche Apps mit Bezug zu Ihrer Gesundheit haben Sie aktuell installiert?

____

Insgesamt betrachtet, wie häufig verwenden Sie diese Apps?

- ◯ Täglich
- ◯ Fast täglich
- ◯ Mehrmals pro Woche
- ◯ Einmal pro Woche
- ◯ Einmal pro Monat
- ◯ Seltener
- ◯ Ich nutze keine Gesundheitsapps
- ◯ Keine Angabe

Wie häufig nutzen Sie diese Apps für folgendes:

	Täglich	Fast täglich	Mehrmals pro Woche	Einmal pro Woche	Seltener	Nie	Keine Angabe
Zum Aufzeichnen von medizinischen Messwerten (z.B. Blutdruck, Herzraten, etc.)	◯	◯	◯	◯	◯	◯	◯
Zum Aufzeichnen von gesundheitsbezogenen Daten (z.B. Sport, Schritte, Ernährung, etc.)	◯	◯	◯	◯	◯	◯	◯
Zur Unterstützung bei der regelmäßigen Medikamenteneinnahme	◯	◯	◯	◯	◯	◯	◯
Zum Zugriff auf Patientenportale (z.B. Terminvereinbarungen, Abruf von Ergebnissen, etc.)	◯	◯	◯	◯	◯	◯	◯
Zum Einsehen von persönlichen Gesundheitsdaten (elektronische Patientenakte)	◯	◯	◯	◯	◯	◯	◯
Für Kontakt zu Experten (z.B. ÄrztInnen, etc.)	◯	◯	◯	◯	◯	◯	◯
Für Kontakt zu anderen Patienten	◯	◯	◯	◯	◯	◯	◯

Wurden Sie vor der Appnutzung angemessen über den Nutzen aufgeklärt?

- ◯ Nein
- ◯ Ja
- ◯ Keine Angabe

Wurden Sie vor der Appnutzung angemessen über die Risiken aufgeklärt?

- ◯ Nein
- ◯ Ja
- ◯ Keine Angabe

Bewerten Sie die von Ihnen genutzten Gesundheitsapps insgesamt in Bezug auf folgende Dimensionen:

Nicht effektiv	◯	◯	◯	◯	◯	Effektiv
Nicht hilfreich	◯	◯	◯	◯	◯	Hilfreich
Nicht funktional	◯	◯	◯	◯	◯	Funktional
Unnötig	◯	◯	◯	◯	◯	Notwendig
Unpraktisch	◯	◯	◯	◯	◯	Praktisch
Nicht unterhaltsam	◯	◯	◯	◯	◯	Unterhaltsam
Langweilig	◯	◯	◯	◯	◯	Aufregend
Nicht reizvoll	◯	◯	◯	◯	◯	Reizvoll
Nicht spannend	◯	◯	◯	◯	◯	Spannend
Unangenehm	◯	◯	◯	◯	◯	Angenehm

Welchen Nutzen sehen Sie persönlich in Bezug auf Ihre Brustkrebserkrankung durch Gesundheitsapps?

Welche Risiken sehen Sie persönlich in Bezug auf Ihre Brustkrebserkrankung durch Gesundheitsapps?

Bitte geben Sie an, wie sie zu folgenden Aussagen stehen:

	Sehr unwahrscheinlich	Unwahrscheinlich	Neutral	Wahrscheinlich	Sehr wahrscheinlich
Gesundheitsapps ermöglichen es mir, besser darüber informiert zu sein, wie ich die Ratschläge von Ärzten oder Fachleuten, befolgen kann.	○	○	○	○	○
Die Nutzung von Gesundheitsapps ermöglicht es mir, ein besseres Verständnis für meine persönliche Gesundheit zu entwickeln.	○	○	○	○	○
Gesundheitsapps tragen dazu bei, dass ich mich besser in der Lage fühle, die Ratschläge von Ärzten oder Fachleuten umzusetzen.	○	○	○	○	○
Durch die Nutzung von Gesundheitsapps fühle ich mich sicherer, eine aktivere Rolle im Austausch mit meinem Arzt oder anderen Fachleuten zu spielen.	○	○	○	○	○
Die Nutzung von Gesundheitsapps ermöglicht es mir, selbstständige Entscheidungen über meine Gesundheit zu treffen.	○	○	○	○	○

digiOnko

Erleben Sie in ihrem Alltag Nachteile und/oder Diskriminierung aufgrund persönlicher Merkmale, die ihr Geschlecht, ihre Hautfarbe, ihre Nationalität, ihr Einkommen oder das Zusammentreffen dieser Kategorien betreffen?

- ◯ Nein
- ◯ Ja
- ◯ Keine Angabe

Befürchten Sie persönliche Nachteile und/oder Diskriminierung durch eine Appnutzung aufgrund persönlicher Merkmale, die ihr Geschlecht, ihre Hautfarbe, ihre Nationalität, ihr Einkommen oder das Zusammentreffen dieser Kategorien betreffen?

- ◯ Nein
- ◯ Ja
- ◯ Keine Angabe

Gibt es sonstige Lebensumstände, die Sie an der Nutzung einer Gesundheitsapp in ihrem Alltag möglicherweise hindern?

- ◯ Ja, folgende: ______________________________
- ◯ Nein
- ◯ Keine Angabe

digiOnko

Bitte geben Sie an, wie sie zu folgenden Fragen stehen:

	Sehr unwahrscheinlic h	Unwahrscheinlic h	Neutral	Wahrscheinlich	Sehr wahrscheinlich
Würden Sie Ihr Smartphone benutzen wollen, um Ihre krebsbezogenen Informationen über eine App auf Ihrem Smartphone zu verfolgen?	◯	◯	◯	◯	◯
Würden Sie eine App auf Ihr Smartphone herunterladen, um Ihr Wissen über Krebs zu erweitern?	◯	◯	◯	◯	◯
Wären Sie bereit, täglich eine App auf Ihrem Smartphone zu verwenden, die Ihnen hilft, Ihren Gesundheitszustand zu beobachten?	◯	◯	◯	◯	◯

Welche Funktionen erwarten Sie in einer Gesundheitsapp die PatientInnen mit Brustkrebs unterstützt?

Bitte geben Sie an, wie Sie zu folgenden Aussagen stehen:

Ich würde meine anonymisierten Gesundheitsdaten eher der medizinischen Forschung zur Verfügung stellen, wenn…

	Sehr unwahrscheinlich	Unwahrscheinlich	Neutral	Wahrscheinlich	Sehr wahrscheinlich
... ich dafür Geld erhalten würde.	◯	◯	◯	◯	◯
... dadurch die Gesundheitskosten und damit langfristig Krankenversicherungsbeiträge sinken würden.	◯	◯	◯	◯	◯
... andere Leute dadurch eine bessere Behandlung erhalten würden.	◯	◯	◯	◯	◯
... ich genau wüsste, für welche konkrete Forschung meine Daten verwendet würden.	◯	◯	◯	◯	◯
... ich sicher sein könnte, dass die Daten vor Missbrauch genügend geschützt werden.	◯	◯	◯	◯	◯
... ich die Daten in einem Gesundheitsportal online selbst verwalten könnte.	◯	◯	◯	◯	◯
... eine vertrauenswürdige Insititution die Daten in einem Gesundheitsportal online für mich verwalten würde.	◯	◯	◯	◯	◯
... ich meine Zustimmung zur Datennutzung jederzeit zurückziehen könnte.	◯	◯	◯	◯	◯

Bitte geben Sie an, wie Sie zu folgenden Aussagen stehen:

Ich würde meine anonymisierten Gesundheitsdaten der medizinischen Forschung zur Verfügung stellen, wenn…

	Sehr unwahrscheinlich	Unwahrscheinlich	Neutral	Wahrscheinlich	Sehr wahrscheinlich
... trotz Anonymisierung jemand Rückschlüsse auf meine Person machen könnte.	○	○	○	○	○
... die Krankenversicherung meine Daten analysieren kann.	○	○	○	○	○
... andere Leute oder Unternehmen finanziell von meinen Daten profitieren.	○	○	○	○	○
... ich persönlich keinen direkten gesundheitsheitlichen Nutzen davon habe.	○	○	○	○	○
.... ich das Gefühl habe, dass meine Privatsphäre nicht genug geschützt ist.	○	○	○	○	○

Aus welchen Gründen möchten Sie Ihre Daten teilen bzw. nicht teilen?

____

Sie haben angekreuzt, dass sie ein Gesundheitsportal befürworten, in dem Sie oder eine Institution Ihre Daten verwaltet.

Welche Anforderungen haben Sie an ein solches Gesundheitsportal?

____

Würden Sie Geld zahlen, um ihre Daten von einer vertrauenswürdigen Institution verwalten zu lassen?

- ◯ Nein
- ◯ Ja
- ◯ Keine Angabe

Sie beschreiben sich als...

- ◯ Weiblich
- ◯ Männlich
- ◯ Divers
- ◯ Keine Angabe

In welchem Jahr haben Sie Ihre Brustkrebsdiagnose erhalten?

- ◯ Jahr: ____________________
- ◯ Weiß nicht
- ◯ Keine Angabe

Erhalten Sie aktuell ein Behandlung für Ihren Brustkrebs oder befinden Sie sich in der Nachsorge?

- ◯ In Behandlung
- ◯ In der Nachsorge
- ◯ Weiß nicht
- ◯ Keine Angabe

Wurden bei Ihnen Metastasen festgestellt?

◯ Nein

◯ Ja

◯ Weiß nicht

◯ Keine Angabe

Was ist Ihr höchster Bildungsabschluss?

◯ Kein Abschluss

◯ Haupt-/Mittelschulabschluss

◯ Realschulabschluss

◯ Abitur

◯ Berufsausbildung

◯ Hochschulabschluss

◯ Promotion

◯ Sonstige

◯ Keine Angabe

Wie ist Ihr momentaner beruflicher Status?

◯ Vollzeit erwerbstätig

◯ Teilzeit erwerbstätig

◯ Geringfügig beschäftigt (520€ Job)

◯ Selbstständig/Freiberuflich

◯ Rente/Pension

◯ Elternzeit

◯ Weiterbildung

◯ Nicht erwerbstätig

◯ Arbeitslos/Arbeitssuchend

◯ Sonstiges

◯ Keine Angabe

Wie hoch ist Ihr monatliches Netto-Haushaltseinkommen?

◯ Weniger als 1.000 Euro pro Monat

◯ 1.000 bis 2.000 Euro pro Monat

◯ 2.000 bis 3.000 Euro pro Monat

◯ Mehr als 3.000 Euro pro Monat

◯ Keine Angabe

Wie sind Sie krankenversichert?

◯ Gesetzlich

◯ Privat

◯ Nicht versichert

◯ Weiß nicht

◯ Keine Angabe

Was entspricht am ehesten Ihrem aktuellen Wohnort?

◯ In der Stadt

◯ Am Stadtrand

◯ In einem Vorort

◯ Eher Ländlich

◯ Auf dem Land

◯ Sonstiges

◯ Keine Angabe

Wie viele Erwachsene (über 18 Jahren) leben in Ihrem Haushalt?

◯ Anzahl:

____

◯ Keine Angabe

digiOnko

Wie viele Kinder (unter 18 Jahren) leben in Ihrem Haushalt?

◯ Anzahl: ____________________

◯ Keine Angabe

Gibt es noch etwas, das Sie uns mitteilen möchten?

____
