# Supplementary material for: Acceptance, Perceived Usefulness, and Data Sharing in Mobile Health Apps Among Patients With Breast Cancer: Cross-Sectional Survey Study
Source: JMIR Cancer. 2026 Apr 7;12:e77750. doi: 10.2196/77750 (PMC13056039; doi:10.2196/77750)
Supplement: Multimedia Appendix 2 [file cancer-v12-e77750-s002.pdf]

digiOnko

Which year were you born?

____

Have you ever been diagnosed with breast cancer?

- ◯ No
- ◯ Yes

Do you own a smartphone?

- ◯ No
- ◯ Yes
- ◯ Prefer not to say

Do you own a tablet?

- ◯ No
- ◯ Yes
- ◯ Prefer not to say

Do you own a smartwatch or a fitnesstracker?

- ◯ No
- ◯ Yes
- ◯ Prefer not to say
- ◯ I don't know what that is

How often do you go online?

- ◯ Daily
- ◯ Almost daily
- ◯ Several times a week
- ◯ Once a week
- ◯ Less frequently
- ◯ Prefer not to say

How confident do you feel when using your smartphone and apps?

- ◯ Very insecure
- ◯ Rather insecure
- ◯ Neutral
- ◯ Rather secure
- ◯ Very secure
- ◯ I don't use a smartphone

How often do you use your smartphone?

- ◯ Daily
- ◯ Almost daily
- ◯ Several times a week
- ◯ Once a week
- ◯ Less frequently
- ◯ Prefer not to say

How often do you use your tablet?

- ◯ Daily
- ◯ Almost daily
- ◯ Several times a week
- ◯ Once a week
- ◯ Less frequently
- ◯ Prefer not to say

How often do you wear your smartwatch or your fitnesstracker?

- ◯ Daily
- ◯ Almost daily
- ◯ Several times a week
- ◯ Once a week
- ◯ Less frequently
- ◯ Prefer not to say

Have you used any of the following resources to improve your health status in the past 12 months?

	No	Yes	Prefer not to say
Audiobooks and Podcasts	◯	◯	◯
Social Media (e.g., Facebook, Instagram, etc.)	◯	◯	◯
Video-Calls	◯	◯	◯
Apps for smartphone and/or Tablets	◯	◯	◯
Printed materials (e.g., flyer,books, etc.)	◯	◯	◯
Internet (e.g., Google)	◯	◯	◯

Which health-related apps are currently installed on your device?

____

Overall, how often do you use these apps?

- ◯ Daily
- ◯ Almost daily
- ◯ Several times a week
- ◯ Once a week
- ◯ Once a month
- ◯ Less frequently
- ◯ I do not use any health-related apps
- ◯ Prefer not to say

How often do you use these apps for the following:

	Daily	Almost daily	Several times a week	Once a week	Less frequently	Never	Prefer not to say
For recording medical measurements (e.g., blood pressure, heart rate, etc.)	○	○	○	○	○	○	○
For recording health-related data (e.g., workout, steps, nutrition, etc.)	○	○	○	○	○	○	○
To help with regular medication intake	○	○	○	○	○	○	○
To access patient portals (e.g., scheduling appointments, retrieving test results, etc.)	○	○	○	○	○	○	○
To view personal health data (electronic patient record)	○	○	○	○	○	○	○
To contact experts (e.g., doctors, etc.)	○	○	○	○	○	○	○
To contact other patients	○	○	○	○	○	○	○

digiOnko

Were you adequately informed about the benefits before using the app?

- ◯ No
- ◯ Yes
- ◯ Prefer not to say

Were you adequately informed about the risks before using the app?

- ◯ No
- ◯ Yes
- ◯ Prefer not to say

Please rate the health apps you use overall in terms of the following dimensions:

Not effective	◯	◯	◯	◯	◯	Effective
Not helpful	◯	◯	◯	◯	◯	Helpful
Not functional	◯	◯	◯	◯	◯	Functional
Unneccessary	◯	◯	◯	◯	◯	Necessary
Impractical	◯	◯	◯	◯	◯	Practical
Not fun	◯	◯	◯	◯	◯	Fun
Dull	◯	◯	◯	◯	◯	Exciting
Not delightful	◯	◯	◯	◯	◯	Delightful
Not thrilling	◯	◯	◯	◯	◯	Thrilling
Enjoyable	◯	◯	◯	◯	◯	Unenjoyable

What benefits do you personally see in health apps in relation to your breast cancer?

What risks do you personally see in health apps in relation to your breast cancer?

---

digiOnko

Please indicate your opinion on the following statements:

	Very unlikely	Unlikely	Neutral	Likely	Very likely
Health apps enable me to be better informed about how to follow the advice of doctors or other professionals.	◯	◯	◯	◯	◯
Using health apps enables me to develop a better understanding of my personal health.	◯	◯	◯	◯	◯
Health apps ar helping me better follow the advice of doctors or other professionals.	◯	◯	◯	◯	◯
Using health apps makes me feel more confident about playing a more active role in communicating with my doctor or other professionals.	◯	◯	◯	◯	◯
Using health apps enables me to make independent decisions about my health.	◯	◯	◯	◯	◯

Do you experience disadvantages and/or discrimination in your everyday life due to personal characteristics related to your gender, skin color, nationality, income, or a combination of these categories?

- ◯ No
- ◯ Yes
- ◯ Prefer not to say

Are you concerned about personal disadvantages and/or discrimination when using a health app due to personal characteristics relating to your gender, skin color, nationality income or a combination of these categories?

- ◯ No
- ◯ Yes
- ◯ Prefer not to say

Are there any other reasons that might prevent you from using a health app in your everyday life?

- ◯ Yes, the following: ______________________________
- ◯ No
- ◯ Prefer not to say

digiOnko

Please indicate your opinion on the following questions:

	Very unlikely	Unlikely	Neutral	Likely	Very likely
Would you want to use your smartphone to track your cancer-related information via an app on your smartphone	○	○	○	○	○
Would you download an app to your phone to expand your knowledge about cancer?	○	○	○	○	○
Would you be willing to use an app on your phone every day that helps you monitor your health status?	○	○	○	○	○

What features do you expect in a health app that support patients with breast cancer?

digiOnko

Please indicate your opinion on the following statements:

I would be more willing to make my anonymized health data available for medical research if...

	Very unlikely	Unlikely	Neutral	Likely	Very likely
... I would receive money for it.	○	○	○	○	○
... if healthcare costs and with that in the long-term health insurance costs would decrease.	○	○	○	○	○
... other people would receive better therapy because of it.	○	○	○	○	○
... I knew exactly what specific research my data would be used for.	○	○	○	○	○
... I could be sure, that my data is protected sufficiently against misuse.	○	○	○	○	○
... I could manage the data myself on a health portal.	○	○	○	○	○
... a trustworthy institution would manage my data online in a health portal.	○	○	○	○	○
... I could withdraw my consent to the use of my data at any time.	○	○	○	○	○

Please indicate your opinion on the following statements:

I would be willing to make my anonymized health data available for medical research if...

	Very unlikely	Unlikely	Neutral	Likely	Very likely
... despite anonymization someone could draw conclusions about my identity.	◯	◯	◯	◯	◯
... the health insurance could analyse my data.	◯	◯	◯	◯	◯
... oder people or companies would benefit financially from my data.	◯	◯	◯	◯	◯
... I personally do not have any direct health benefits from it.	◯	◯	◯	◯	◯
.... I feel that my privacy is not sufficiently protected.	◯	◯	◯	◯	◯

For what reasons would you like to share or not share your data?

____

You have indicated that you support a health portal where you or an institution manages your data.

What requirements do you have for such a health portal?

____

Would you pay to have your data managed by a trusted institution?

- ◯ Nein
- ◯ Ja
- ◯ Prefer not to say

You identify as...

- ◯ Female
- ◯ Male
- ◯ Diverse
- ◯ Prefer not to say

In what year were you diagnosed with breast cancer?

- ◯ Year: ____________________
- ◯ I don't know
- ◯ Prefer not to say

Are you currently receiving treatment for your breast cancer or are you in follow-up care?

- ◯ In treatment
- ◯ In follow-up care
- ◯ I don't know
- ◯ Prefer not to say

Have you been diagnosed with metastases?

◯ No
◯ Yes
◯ I don't know
◯ Prefer not to say

What is your highest level of education?

◯ No degree
◯ High school/middle school diploma
◯ Secondary school diploma
◯ A levels
◯ Vocational training
◯ University degree
◯ Doctorate
◯ Others
◯ Prefer not to say

What is your current professional status?

◯ Full-time employed
◯ Part-time employed
◯ Minor employment (520€ job)
◯ Self-employed/Freelance
◯ Retired
◯ Parental leave
◯ Further education
◯ Not employed
◯ Unemployed/seeking employment
◯ Others
◯ Prefer not to say

What is your monthly net household income?

- ◯ Less than 1.000 Euro a month
- ◯ 1.000 to 2.000 Euro a month
- ◯ 2.000 bis 3.000 Euro a month
- ◯ More than 3.000 Euro a month
- ◯ Prefer not to say

What kind of health insurance do you have?

- ◯ Statutory
- ◯ Private
- ◯ Not insured
- ◯ I don't know
- ◯ Prefer not to say

Which of the following best describes your current place of residence?

- ◯ Urban
- ◯ On the outskirts
- ◯ Suburban
- ◯ More rural
- ◯ Rural
- ◯ Others
- ◯ Prefer not to say

How many adults (over 18 years of age) live in your household?

- ◯ Number: ____________________
- ◯ Prefer not to say

digiOnko

How many children (under 18 years of age) live in your household?

◯ Number:

____

◯ Prefer not to say

Anything else you would like to tell us?

____
